# LiDAR Scan Matching Aided Inertial Navigation System in GNSS-Denied Environments

**DOI:** 10.3390/s150716710

**Published:** 2015-07-10

**Authors:** Jian Tang, Yuwei Chen, Xiaoji Niu, Li Wang, Liang Chen, Jingbin Liu, Chuang Shi, Juha Hyyppä

**Affiliations:** 1 GNSS Research Center, Wuhan University, 129 Luoyu Road, Wuhan 430079, Hubei, China; E-Mails: tangjian@whu.edu.cn (J.T.); xjniu@whu.edu.cn (X.N.); li.wang@whu.edu.cn (L.W.); shi@whu.edu.cn (C.S.); 2 Department of Remote Sensing and Photogrammetry, Finnish Geospatial Institute, Kirkkonummi FI-02431, Finland; E-Mails: jingbin.liu@nls.fi (J.L.); juha.hyyppa@nls.fi (J.H.); 3 Department of Navigation and Positioning, Finnish Geospatial Research Institute, Geodeetinrine 2, Kirkkonummi FI-02431, Finland; E-Mail: liang.chen@nls.fi

**Keywords:** LiDAR, scan matching, INS, EKF, inertial navigation

## Abstract

A new scan that matches an aided Inertial Navigation System (INS) with a low-cost LiDAR is proposed as an alternative to GNSS-based navigation systems in GNSS-degraded or -denied environments such as indoor areas, dense forests, or urban canyons. In these areas, INS-based Dead Reckoning (DR) and Simultaneous Localization and Mapping (SLAM) technologies are normally used to estimate positions as separate tools. However, there are critical implementation problems with each standalone system. The drift errors of velocity, position, and heading angles in an INS will accumulate over time, and on-line calibration is a must for sustaining positioning accuracy. SLAM performance is poor in featureless environments where the matching errors can significantly increase. Each standalone positioning method cannot offer a sustainable navigation solution with acceptable accuracy. This paper integrates two complementary technologies—INS and LiDAR SLAM—into one navigation frame with a loosely coupled Extended Kalman Filter (EKF) to use the advantages and overcome the drawbacks of each system to establish a stable long-term navigation process. Static and dynamic field tests were carried out with a self-developed Unmanned Ground Vehicle (UGV) platform—NAVIS. The results prove that the proposed approach can provide positioning accuracy at the centimetre level for long-term operations, even in a featureless indoor environment.

## 1. Introduction

High-precision dynamic positioning is in great demand in Unmanned Aerial Vehicle (UAV) and Unmanned Ground Vehicle (UGV) applications. The most popular technology is the integration of carrier-phase-based differential global navigation satellite systems (GNSS) and commercial-grade or tactical-grade Inertial Navigation Systems (INS) to provide centimetre-level high-accuracy navigation with good GNSS availability [1]. However, a GNSS signal cannot be always available; in a GNSS-denied environment, INS can offer an accurate position solution for a short period, although drift will accumulate and errors will increase without bounds.

Various methods to reduce or bound INS drift have been addressed in the past. A feature-aided INS with external sensors can be placed in odometers, magnetometers, or cameras for Light Detection and Ranging (LiDAR) as an alternative to satellite-based navigation technology in a GNSS-denied environment. The most popular feature-aided INS solutions are visually aided and LiDAR-aided systems [2]; they offer affordable solutions that are not affected by RF signal blockage. To achieve a high level of positioning estimation in a close-range area, a visual-aided system uses a texture feature that matched consecutive images that are captured by a calibrated camera. Various feature extraction and matching methods and algorithms based on a passive sensing solution have been developed in previous works: a stochastic projection was proposed by Veth, Michael J. to track features [3,4]; Scale-Invariant Feature Tracking (SIFT), SURF, Center Surround Extrema (CenSURE), and RANSAC algorithms were introduced in a visual odometry application for feature tracking by Scaramuzza & Fraundorfer [5]; and a Hough Transformation (HT) and photogrammetric algorithms have been frequency adopted in feature tracking [6]. Moreover, Inertial Measurement Units (IMU) have been applied in some monocular camera-based visual SLAM (Simultaneous Localization and Mapping) systems, primarily for attitude estimation [7,8,9,10]. Though SLAM systems focus on localization and mapping, the essence of feature matching in SLAM is identical to visually based navigation systems. However, this passive sensing solution extensively relies on the lighting situation of the detected environment, which restricts its applications. Conversely, LiDAR is an active ranging sensor with a laser source that can be used in environments where natural or artificial light sources are not available. 

Most existing LiDAR-aided INSs currently use similar positioning methods with a visually aided system; they extract geometric features (points, lines, or planes) from laser scans [11,12,13,14,15,16,17], which increase computing complexity and decrease system stability and availability. Stefan *etc.* carried out pilot research by proposing a LiDAR scan matching method with a Gauss-Newton algorithm; the matching results were fused with the measurements of an IMU to estimate a full 3D-motion of a moving platform. It is now available as an open-source package for Robot Operating Systems (ROS) [12]; the accuracy of the proposed method is unknown. GMapping has also been widely adopted by various indoor mapping/SLAM programs that use long-range raw LiDAR range data and odometry. However, it has not been optimized for short-range laser scanners, and accurate odometry measurements are absent from most low-cost platforms [18]. As already discussed in [19], the position of some fine-featured objects cannot actually be precisely measured due to the footprint size of the deployed laser scanner. This means that the measured coordinates of the geometry features already contain measurement errors, and these errors will propagate into the final mapping and positioning results. 

Feature-aided inertial navigation can be divided into two categories: relative-based systems and absolute-based systems (also called optic flow and feature- or landmark-based visual navigation). Features in consecutive images or scans are detected and relatively matched to determine the rigid-body transformation (translation and rotation) of the sensing platform [4,15,20]. A photogrammetry method is applied while in absolute navigation to rectify position and attitude [11,20] on the premise that the positions of landmarks detected from the images or scans in a global coordinate reference system are already known. After the system is fused with IMUs, it is able to overcome the inherent limitations and drawbacks of each standalone system. This study assumes that the system can work in an unknown environment, and a relative feature-aided method is thus investigated.

Previous research has shown that an Extended Kalman Filter (EKF) is a relatively robust and efficient error estimating framework suitable for dynamic motion systems [21]; in particular, it is widely used in visually based SLAM and navigation systems [6,7,8,9,10,11]. Thus, the EKF is used in this research on the fusion of IMU and LiDAR scan matching.

A global scan matching aided INS method is thus proposed to establish an efficient navigation system for GNSS denied environment; the entire system consists of 2D LiDAR and a commercial-grade IMU sensor. The low-cost IMU provides a short-term coarse transformation of position and attitude. A 2D laser scanner with a self-developed improved probabilistically motivated Maximum Likelihood Estimation (IMLE) algorithm [22] then uses these transformations to refine the search scope to estimate an accurate position and attitude. Finally, these standalone positioning results are loosely coupled with EKF to obtain the final result. Compared with existing LiDAR-aided INS positioning solutions, this paper offers several major contributions. First, a non-feature extracted-grid map-based global scan matching algorithm is applied to aid the inertial system. It is more accurate and stable while providing low computational complexity. Second, the IMLE algorithm is a brute global optimum search method, and an IMU sensor is used to provide an accurate initial position and narrow search scope that can assure the IMLE algorithm avoids a local optimum and accelerates the computation for subsequent real-time applications. Third, LiDAR scan matching depends heavily on environmental features, and IMUs can assist system navigation in a featureless “outage” environment for a short period to sustain a highly accurate positioning solution until geometric features are detected to aid the inertial system. Finally, the fused result can also rectify the initial state of position, velocity and attitude of the INS to sustain long-term running applications.

The rest of this paper is organized as follows: Section 2 describes the system workflow and error models of the INS and LiDAR; Section 3 discusses the field tests and experimental results; and Section 4 offers conclusions.

## 2. INS and LiDAR Fusion Modelling 

### 2.1. INS Modelling

The INS navigation frame (n-frame) is a local geodetic frame with the x-axis pointing towards geodetic north, the z-axis orthogonal to the reference ellipsoid pointing down, and the y-axis completing a right-handed orthogonal frame. It is also called a north-east-down (NED) system. The body frame (b-frame) is defined at the IMU centre. The dynamic equations in the n-frame are given by [23,24]:

(1) $${\dot{r}}^{n}=D^{-1}v^{n}$$

(2) $${\dot{v}}^{n}=C_{b}^{n}\left(f^{b}-b_{a}\right)-\left(2\omega_{ie}^{n}+\omega_{en}^{n}\right)v^{n}+g^{n}$$

(3) $${\dot{C}}_{b}^{n}=C_{b}^{n}\left(\omega_{nb}^{b}\times \right)$$

(4) $$\omega_{nb}^{b}=\;\omega_{ib}^{b}-C_{n}^{b}\left(\omega_{ie}^{n}+\omega_{en}^{n}\right)-b_{g}$$

(5) $$D^{-1}=\left[\begin{array}{ccc} 1/\left(M+h\right) & 0 & 0\\ 0 & 1/\left(N+hcos\text{φ}\right) & 0\\ 0 & 0 & -1 \end{array}\right]$$

where the position in the navigation frame is $r^{n}=\left[\text{φ},\text{λ},\text{h}\right]$, φ is the latitude, λ is the longitude, and h is the height above the earth surface; $v^{n}=\left[v_{N},v_{E},v_{D}\right]$ is the platform velocity; $C_{n}^{b}$ is the transformation matrix from the b-frame to the n-frame and vice-versa for $C_{b}^{n}$; $f^{b}$ is the specific force; $\omega_{ib}^{b}$ is the body angular rate measured by gyroscopes expressed in the b-frame; $\omega_{ie}^{n}$ and $\omega_{en}^{n}$ are the Earth turn rate in n-frame and the turn rate of the n-frame with the respect to the Earth; ($\;\omega_{nb}^{b}\times$) is the skew symmetric matrix of $\omega_{nb}^{b}$; $g^{n}$ is the local gravity vector; M and N are the radii of curvature in the meridian and prime vertical; and $b_{a}\;and\;b_{g}$ are the drift of the accelerometer and gyroscope, respectively.

If there were no additional errors, the above mechanization equations could estimate the position and velocity of the system from the raw data of the IMU. However, the IMU outputs contain errors and cause the navigation results to rapidly drift; compensating for this drift is difficult. Thus, an error propagation model must work alongside the system motion model to further correct and obtain a better navigation solution; this is also the theoretic basis of the INS and LiDAR EKF fusion model of this paper. A classic perturbation analysis via a first-order Taylor series expansion is applied, and the error state vector and state-space model are defined as follows:

(6) $$\text{δ}x=\left[\text{δ}r^{n},\text{δ}v^{n},{\text{ε}}^{n},\text{δ}b_{a},\text{δ}b_{g}\right]$$

(7) $$\text{u}=\left[\text{ δ}f^{b},\text{δ}\omega_{ib}^{b}\right]$$

(8) $$\text{δ}\dot{x}=F\text{δ}x+\text{Gu}$$

(9) $$\delta x_{k+1}=\emptyset_{k}\text{δ}x_{k}+w_{k}$$

(10) $$\emptyset_{k}=\exp\left(F\text{∆}t\right)\approx I+F\text{∆}t$$

(11) $$\text{Q}=\text{diag}\left(\delta_{g}^{2},\delta_{a}^{2}\right)$$

(12) $$Q_{k}\approx \emptyset_{k}GQG^{T}\emptyset_{k}^{T}\text{∆}t$$

where the error state δx are errors of position ($\delta r^{n}$), velocity ($\delta v^{n}$), attitude ($\varepsilon^{n}$), accelerometer ($\delta b^{a}$), and gyroscope drift ($\delta b^{g}$); F is the dynamic matrix, G is a design matrix and u is the forcing vector of white noise, according to the system motion model and concrete formation of F, G that can be found in the works of *Shin*, *2001* and *2005* [25,26]; $\emptyset_{k}$ is the state transition matrix and $w_{k}$ is the driven response of the input white noise at time $t_{k+1}$, *i.e.*, $w_{k}\sim \text{N}\left(0,Q_{k}\right)$; $Q_{k}$ is the covariance matrix; Q is the spectral density matrix, and $\delta_{g},\delta_{a}$ are the standard deviations of accelerometers and gyroscopes, respectively. 

### 2.2. LiDAR Scan Matching

LiDAR is an active range measuring sensor with a laser source that can detect the geometric information of the environment. However, most existing LiDAR-aided inertial navigation systems use feature (point, line, or plane) extraction and matching methods for assisted navigation. The workflow is more complicated and the extraction process may eliminate effective matching features. Moreover, this method is unstable and unreliable, particularly in featureless environments. Thus, a full scan-matching aided INS is proposed in this paper.

The proposed LiDAR scan-matching algorithm-IMLE is a probabilistic scan matching method based on the feature uncertainty model of a LiDAR sensor [27,28]. A likelihood map M stores the likelihood value created from the previous laser scans and the incoming new scans $S_{t}$ are then matched against the map to find the best body transformation $T^{*}$, where the entire scan provides the maximum likelihood value $\text{P}(S_{t}|\text{M})$. The likelihood value $\text{P}(p_{i}|\text{M})$ of a single point $p_{i}$ on a map M is proportional to the distance d to the nearest environmental feature F, according to the Gaussian probability model of laser-measured noise with scale parameter σ:

(13) $$\text{P}\left(p_{i}\text{|M}\right)\propto e^{\left(-d\left(p_{i,}F\right)/\sigma \right)}$$

(14) $$\text{P}\left(S_{t}\text{|M}\right)=\;\overset{n}{\underset{i=1}{\prod }}\text{P}\left(p_{i}\text{|M}\right)$$

(15) $$T^{*}=\text{argmax}(\text{P}(\text{T}\propto S_{t}|\text{M}))$$

**Figure 1:**
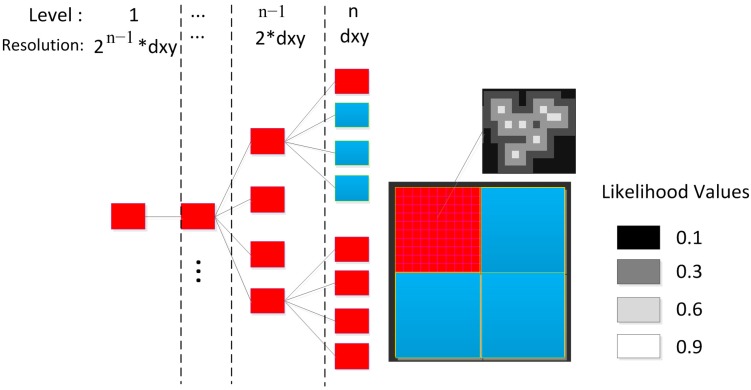
The pyramid structure of likelihood map and the pre-defined likelihood values.

As shown in Figure 1, in an IMLE scan matching model, the likelihood map M is organized as a quad-tree pyramid structure to store the likelihood value with multi-resolutions for a large area. It is geo-projected to the INS navigation frame (n-frame) with a Universal Transverse Mercator (UTM) 35N project coordinate reference to fuse the output of each standalone system into a universal local reference; the map grid cell is populated with a series of pre-defined likelihood values: 0.1, 0.3, 0.6, and 0.9. These are empirical values based on the Gaussian probability model shown in Equation 13.

**Figure 2:**
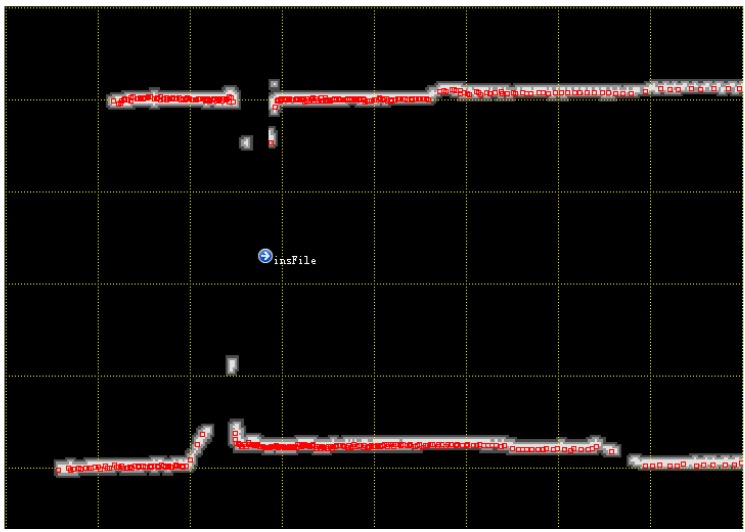
An example of IMLE scan-matching algorithm.

Based on the above model, a brute search algorithm can be deployed to estimate the best body transformation $T^{*}$ within the entire map M. However, this is a time-consuming process; a more practical approach is to search in a refined local search scope extrapolated from the previous state, which can be obtained from the INS. Figure 2 shows an example of an IMLE scan matching algorithm. The red rectangle points indicate the current scan, which searches in the background likelihood map to determine the optimum position and attitude with maximum likelihood values. 

### 2.3. EKF Fusion Modelling

**Figure 3:**
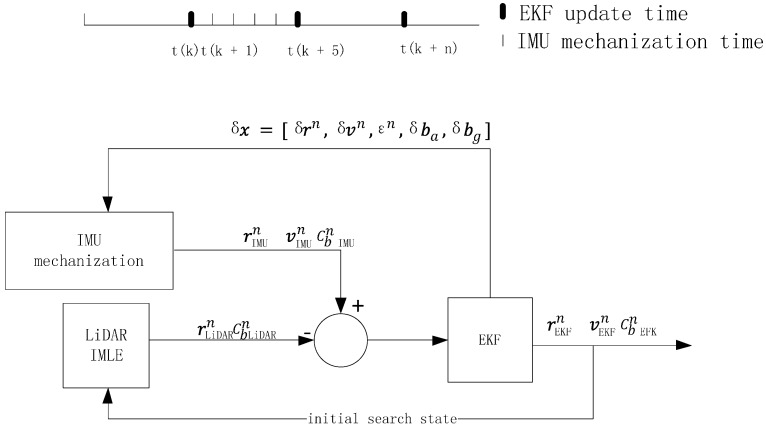
The system architecture of the LiDAR-aided Inertial Navigation System.

An Extended Kalman Filter is selected to fuse the measurements of the INS and LiDAR scan matching; an overview of the system architecture is shown in Figure 3. The Kalman filter algorithm involves predicting the state based on the system model and updating the state based on the measurements [29,30]. However, the output frequency of an IMU is higher than LiDAR measuring. For example, the output rate of an Xsens MTi IMU is approximately 100 Hz, whereas the adopted Hokuyo LiDAR measuring rate is only 40 Hz. Thus, the IMU predicts the state $r_{\text{IMU}}^{n}\;v_{\text{IMU}}^{n}\;C_{b\;\text{IMU}}^{n}$ at every period by mechanization; EKF filters the results only when the periods of LiDAR observation information $r_{\text{LIDAR}}^{n}\;C_{b\;\text{LIDAR}}^{n}$ are obtained. The state error corrections δx are then estimated and fed back to the IMU mechanization for estimating the final navigation state $r_{\text{EKF}}^{n}\;v_{\text{EKF}}^{n}\;C_{b\;\text{EKF}}^{n}$, which will be the initial state for the LiDAR scan-matching search at the next period. The next filter iteration then continues.

The EKF observation functions are as follows:

(16) $$z_{k}=\;H_{k}\delta x_{k}+v_{k}$$

(17) $$z_{k}=\left[\begin{array}{c} r_{IMU}^{n}-r_{LiDAR}^{n}\\ \epsilon_{IMU}^{n}-\epsilon_{LiDAR}^{n} \end{array}\right]$$

(18) $$H_{k}=\left[\begin{array}{cc} \begin{array}{ccc} I_{3\times 3} & 0 & 0\\ 0 & 0 & I_{3\times 3} \end{array} & \begin{array}{cc} 0 & 0\\ 0 & 0 \end{array} \end{array}\right]$$

(19) $$R_{k}=\text{diag}\left(\delta_{r}^{2},\;\delta_{\epsilon }^{2}\right)$$

where $r_{IMU}^{n}$ is the predicted position from the IMU mechanization; $r_{LiDAR}^{n}$ is the observed position from the LiDAR scan matching; $\epsilon_{IMU}^{n}$ and $\epsilon_{LiDAR}^{n}$ are the predicted and observed attitudes, which are expressed as Euler angles, respectively; ${\text{H}}_{\text{k}}$ describes the relation between the state vector and the measurements; ${\text{v}}_{\text{k}}$ is the driven response of the input white noise at time ${\text{t}}_{\text{k}+1}$, *i.e.*, ${\text{v}}_{\text{k}}\sim \text{N}\left(0,{\text{R}}_{\text{k}}\right)$; and ${\text{R}}_{\text{k}}$ is the covariance matrix. ${\text{δ}}_{r}$, ${\text{δ}}_{\epsilon }$ are approximate values based on the properties of the laser scanner device and the angle and range searching intervals of IMLE scan matching.

The estimates of the EKF prediction functions are:

(20) $$\delta x_{k+1}^{-}=\emptyset_{k}\;\delta x_{k-1\;}$$

(21) $$P_{k+1}^{-}=\emptyset_{k}\;P_{k\;}\;{\emptyset_{k}}^{T}\;+\;Q_{k}$$

The Kalman gain is

(22) $$G_{k}=P_{k}^{-}{H_{k}}^{T}S_{k}^{-1}=P_{k}^{-}{H_{k}}^{T}{\left(H_{k}\;P_{k}^{-}\;{H_{k}}^{T}+\;R_{k}\right)}^{-1}$$

The state vector is updated as

(23) $$\delta x_{k}=\delta x_{k}^{-}+\;G_{k}\left(z_{k}-H_{k}\delta x_{k}^{-}\right)$$

(24) $$P_{k}=\left(I-\;G_{k}H_{k}\right)\;P_{k}^{-}$$

where $\delta x_{k}^{-}$ and $P_{k}^{-}$ are the prior estimate and its error covariance.

Finally, the estimated error $\delta x_{k}$ is fed back to the navigation state of position, velocity, and attitude as follows:

(25) $$r_{k}^{n}=r_{k}^{n-}-\;\delta x_{k}^{n}$$

(26) $$v_{k}^{n}=v_{k}^{n-}-\;\delta v_{k}^{n}$$

(27) $$C_{b}^{n}=\left(I+\left(\varepsilon_{k}^{n}\times \right)\right)C_{b}^{n-}$$

(28) $$b_{g}=b_{g}^{-}-\delta b_{g}$$

(29) $$b_{a}=b_{a}^{-}-\delta b_{a}$$

where $r_{k}^{n-}$, $v_{k}^{n-}$, and $C_{b}^{n-}$ are the prior navigation state of position, velocity, and attitude; $\left(\varepsilon_{k}^{n}\times \right)$ is the skew symmetric matrix of attitude error $\varepsilon_{k}^{n}$; and $b_{g}^{-}$ and $b_{a}^{-}$ are the prior drift of the accelerometer and gyroscope.

## 3. Results and Discussion

### 3.1. System Overview

**Figure 4:**
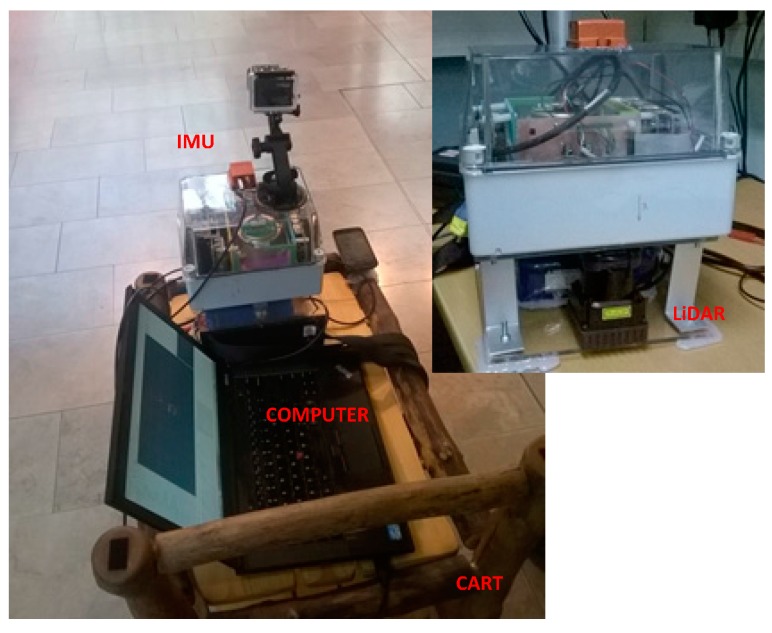
The field test cart platform.

**Figure 5:**
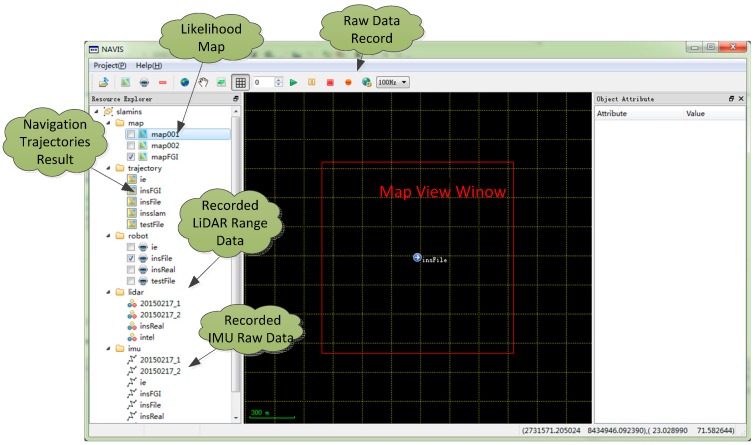
The software platform.

A series of field tests were performed to evaluate the proposed LiDAR-aided inertial navigation system based on [22]. As shown in Figure 4, an Xsens MTi-G IMU and a Hokuyo UTM-30LX-EW laser scanner were installed on a rigid platform and horizontally carried by a cart. They were connected to a laptop with a serial port and a USB port, respectively. The Xsens is a MEMs-based six Degree of Freedom (DOF) miniature commercial grade IMU with an output rate of 100 Hz, an Angular Random Walk (ARW) of 3 $\text{degree}/\sqrt{\text{h}}$, a Velocity Random Walk (VRW) of 0.12 $\text{m}/\text{s}/\sqrt{\text{h}}$, and a Gyro and Accelerometer Bias Instability of 200 degree/h and 2000 mGal (1 Gal = 1 cm/s^2^) [6,31]; The coverage of the LiDAR sensor was approximately 0.1 m to 30 m with a 270° scan angle and an angular resolution of 0.25°. A software platform programmed with C++ and Qt was designed for recording the raw data and post-processing navigation; Figure 5 shows the Graphic User Interface (GUI) of the NAVIS software. 

Two groups of experiments were carried out on the second floor of the Finnish Geospatial Research Institute (FGI) main building: the first group were stationary tests and the second group were dynamic tests along the corridor to evaluate the precision and effectiveness of the position and attitude estimates.

### 3.2. Evaluation of Stationary Estimation

**Figure 6:**
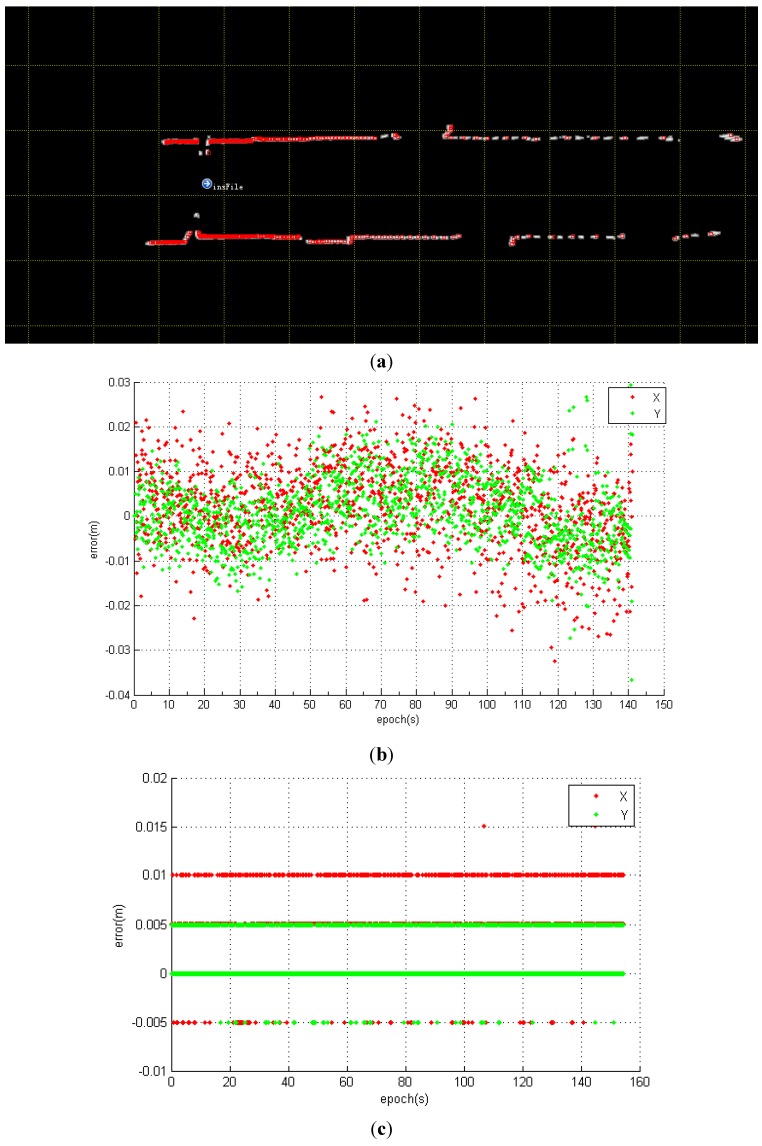

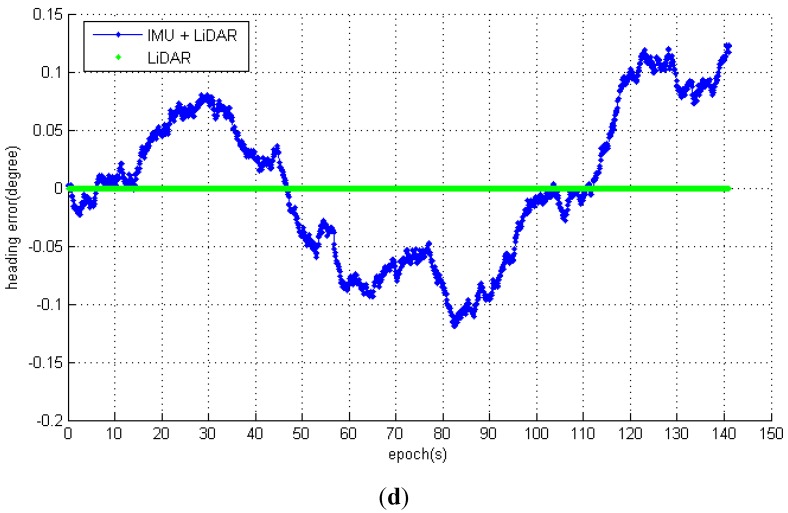
(**a**) Likelihood map result of static filed test. (**b**) The positioning result plot with IMU + LiDAR; (**c**) The positioning result plot with LiDAR scan matching; (**d**) The heading result of IMU + LiDAR and LiDAR scan matching.

**Figure 7:**
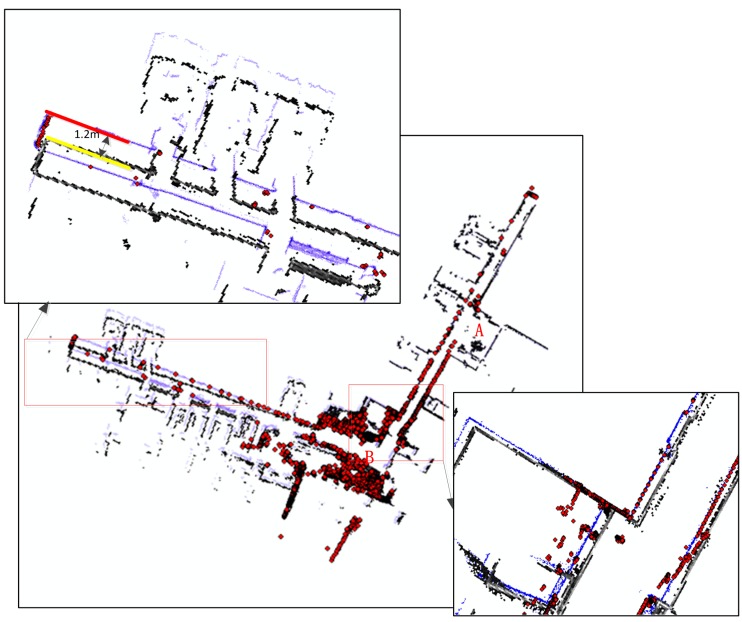
The Mapping results with IMU + LiDAR (blue dot) and LiDAR (black dot), compared with TLS (red dot).

The stationary positioning experiment was performed at the beginning of the corridor for approximately 3 min. The NAVIS was installed on a cart at an installation height of approximately 1.3 m. As seen in Figure 6a, the likelihood map result of the corridor shows a featureless environment where straight parallel walls dominate the scene; Figures 6b,c show the positioning results of the IMU + LiDAR and LiDAR scan matching, respectively. Figure 6d shows the compared heading result with the two different methods. The result plots provide confirmatory evidence for the following conclusions:

1. The error distribution of the LiDAR scan-matching method is stepwise and the error distribution of the IMU + LiDAR resembles white noise. As previously mentioned, the LiDAR scan-matching algorithm is an IMLE, which is a likelihood grid map-based searching method that determines the optimum position from candidates. The likelihood map is divided into small cells as candidate positions according to the map resolution and the angle searching intervals, which are set to 1 cm and 0.25° in this test. We believe this is the primary reason for stepwise error distribution. However, In the IMU + LiDAR combined method, Gaussian error predominates the EKF model, resulting in a white-noise distribution positioning error.
2. The estimated positioning and heading results of LiDAR scan matching are better than the IMU + LiDAR fused method. In the static condition, the incoming laser scan has no feature changes. The LiDAR range measure noise is the only stochastic noise source, and with this optimized condition the IMLE easily detects the platform as stationary. However, when a commercial grade IMU is integrated with LiDAR, the accumulated drift of the gyroscope and accelerometer undermines the accuracy of the final positioning result, although it is verified by LiDAR in an EKF. However, the heading errors are minor and can be neglected for positioning processing. It is anticipated that when a higher-grade IMU (tactical-grade or navigation-grade IMU) is integrated, the positioning error can be mitigated.
3. The positioning results of the y-axis are better than the results of the x-axis, regardless of whether the IMU is integrated. Table 1 shows the numerical statistics of the stationary experiments. The RMS errors of the x-axis, y-axis, and heading estimation with the IMU + LiDAR solution are 0.009 m, 0.007 m, and 0.065°. However, the corresponding RMS errors with the LiDAR only solution are 0.007 m, 0.004 m, and 0.000°. The RMS error of the x-axis is higher than that of the y-axis because there are more features along the y-axis (along the corridor direction) than the x-axis (across the corridor direction) for scan matching. As shown in Figure 7a, almost all laser scan points are horizontally distributed; only a few points are vertically distributed, which makes the positioning accuracy of the Y direction greater than the X direction. This result proves that environmental features proportionally affect positioning results [19]. The reason that the heading estimation equals 0 is that the search step of the current IMLE is 0.25°, with a maximum detected range of 30 m; a 0.25° heading change will cause a maximum 5.3 cm displacement of the laser point on a 30 m target, and this circumstance never occurs during the stationary test.

In stationary positioning, the overall estimated accuracy of position and attitude is higher in the LiDAR scan-matching solution than in the combined IMU + LiDAR solution. The accumulated gyroscope and accelerator drift, as measured by a commercial-grade IMU, deteriorates the final position result, even though the positioning accuracy is still at the centimetre level and the heading estimate RMS error is under 0.2 degrees. The EKF model can be used for LiDAR-aided inertial navigation in certain GNSS-denied environments. This result shows that when an IMU detects a platform as stationary, a LiDAR standalone solution should be deployed rather than the combined solution. 

**Table 1:** The static positioning error statistics (m).

		RMS Error	Mean Error	Maximum Error
IMU + LiDAR	X	0.009	0.007	0.033
	Y	0.007	0.058	0.037
	Heading	0.065 (degree)	0.055 (degree)	0.123 (degree)
LiDAR	X	0.001	0.006	0.015
	Y	0.004	0.003	0.005
	Heading	0.0 (degree)	0.0 (degree)	0.0 (degree)

### 3.3. Evaluation of Dynamic Estimation

To prove the effectiveness of the LiDAR-aided inertial navigation system, further dynamic field tests were performed. The NAVIS was installed on a cart and the cart was driven along the corridor of the third floor of the FGI main building several times by an operator. The results of the IMU + LiDAR combined solution and the LiDAR standalone solution were analysed and compared. 

Figure 7 shows the likelihood map generated with the two different methods. Blue dots represent the map generated with the combined solution; black dots represent the map generated with the LiDAR standalone solution. The two maps are compared with the reference map, which is represented with red dots and generated by a Terrestrial Laser Scanner (TLS). At the beginning of the trajectory, the left sides of the corridors generated with the two methods are aligned and coincide well with the reference point. Then, the map with the black dots begins to deviate from the reference points; the deviation accumulates to approximately 1.2 m at the end of the trajectory. The primary reason for this deviation is the featureless environment at the LiDAR height (1.3 m) consisting of two parallel walls with glass windows and handrails at the small hall (A) and corridor turn (B). Conversely, the blue dots are aligned and coincide well during the entire trajectory. This implies that the estimated errors, inherent in the LiDAR standalone solution, are eliminated by a commercial-grade IMU measurement. These data suggest that the LiDAR-aided inertial system works to mitigate the mapping errors of a LiDAR standalone system. 

It is well known that an inertial navigation system includes both attitude and range estimation. In an IMLE, a normalized $NP(S_{t}|M)$ of LiDAR scan likelihood is introduced to evaluate the navigation quality. It has a relative value from 0 to 1, thus denoting an overlap level of the current scan with previous scans. A value closer to 1 indicates a higher-quality navigation solution. $NP(S_{t}|M)$ can be calculated as follows:

(30) $$\text{NP}({\text{S}}_{\text{t}}|\text{M})\;=\frac{\sum_{\text{i}=1}^{\text{n}}\text{P}\left({\text{p}}_{\text{i}}\text{|M}\right)}{\text{n}}$$

**Figure 8:**
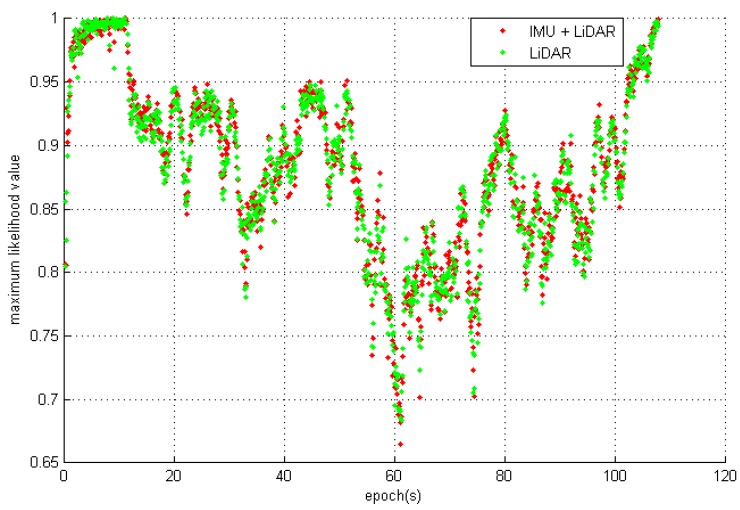
The Maximum Likelihood Value of IMU + LiDAR and LiDAR scan matching.

**Figure 9:**
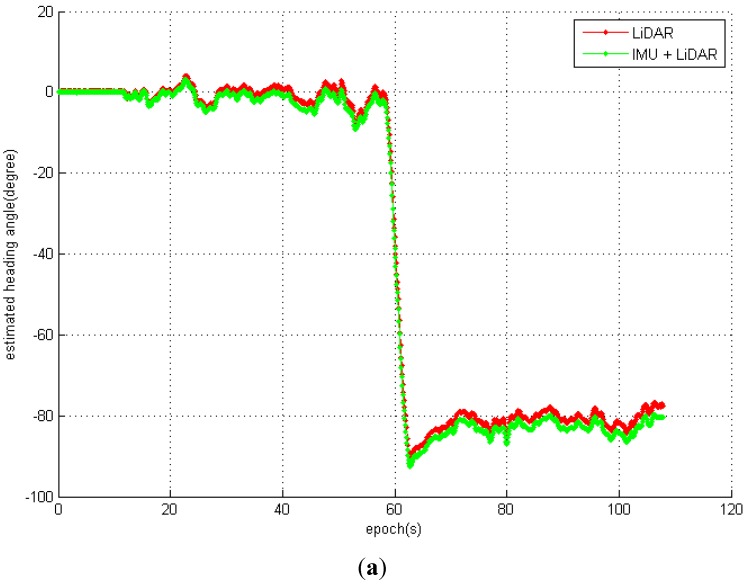

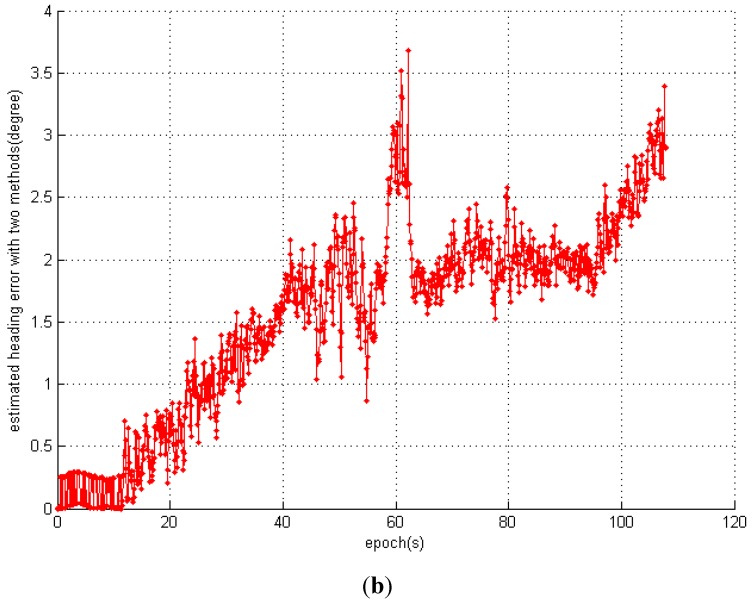
(**a**) The estimated heading of the IMU + LiDAR combined solution and LiDAR standalone solution; (**b**) the estimated heading difference between the IMU + LiDAR combined solution and the LiDAR standalone solution.

A series of $NP(S_{t}|M)$ for each navigation period with the IMU + LiDAR solution and LiDAR standalone solution are shown in Figure 8. The patterns of maximum $NP(S_{t}|M)$ are the same, which implies that the range estimation (and the displacement) is almost identical with two methods. We conclude that the difference in the final trajectories of the two methods is primarily affected by the heading estimation. The main error corrected by IMU is the attitude estimation, and Figure 9a,b show evidence that proves this result. After approximately 15 s, the cart enters the area of the small hall, where there is a relatively feature-poor environment. The heading estimated error appears with the LiDAR standalone solution and the accumulated error does not remain fixed to the end. At 60 s, the heading differences reach a maximum 3.7 degrees at the turn of the corridor, which is full of glass handrails. However, the results also prove that IMUs significantly contribute to attitude estimations, particularly for short-period heading estimations that can sustain an accurate heading estimation in a feature-poor environment for a short period until the LiDAR scan matching re-enters a feature rich environment. 

**Figure 10:**
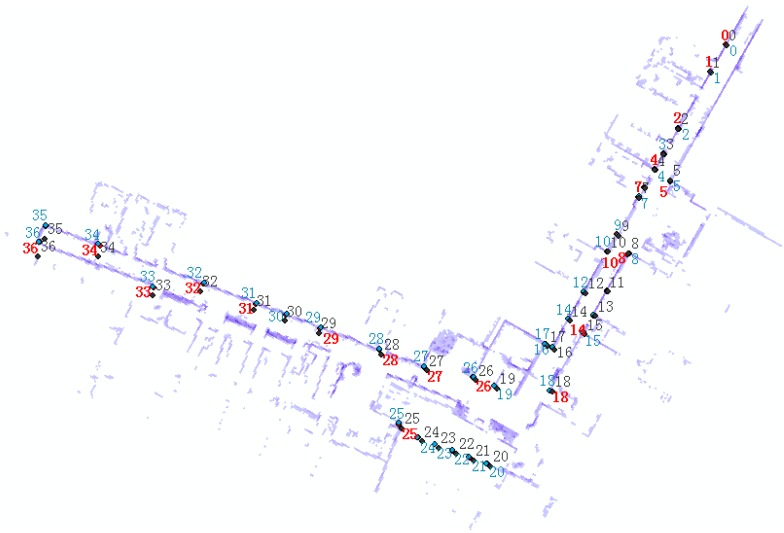
Key points of environmental features selected for accuracy evaluation.

Moreover, 36 key points of environmental features are selected as a reference network to evaluate the mapping and positioning accuracy of the proposed method, as shown in Figure 10. The final accuracy result is shown in Table 2. When the system is moving, the results of the combined solution are obviously better than the LiDAR standalone solution; the RMS error of the combined solution is 0.084 m, remaining at the centimetre level. The RMS error of the other system becomes 0.433 m and drifts to 1.2 m by the end of the trajectory.

**Table 2:** The dynamic positioning error statistics (m).

	RMS Error	Mean Error	Maximum Error
IMU+LiDAR	0.084	0.075	0.188
LiDAR	0.433	0.336	1.195

## 4. Conclusions

In summary, this paper proposes a LiDAR scan-matching aided inertial navigation system based on a commercial-grade IMU and LiDAR combined into one system, with raw IMU outputs used to refine the search scope of SLAM to optimize brute search efficiency. The positioning results calculated by an IMU mechanization algorithm are fused with the SLAM results on a navigation frame with a loosely coupled EKF. This combination mitigates the corresponding inherent drawbacks of a standalone solution to establish stable long-term navigation in a GNSS-denied environment. The results of stationary and dynamic field tests confirm that (1) in a feature poor environment, LiDAR scan matching provides more accurate navigation state estimation than a low-cost IMU + LiDAR system can offer under stationary conditions. In addition, (2) IMU mechanization provides better attitude estimation in dynamic tests, regardless of whether environmental features are rich; it can significantly mitigate the inherent heading estimation errors introduced by scanning matching methods in feature-poor environments. Moreover, (3) LiDAR scan matching contributes to range estimation along the moving direction, which is the basis for accurate heading estimation. Finally, (4) the dynamic positioning accuracy remains at the centimetre level with the proposed combined solution, even in a featureless environment. The proposed LiDAR-aided INS system can overcome the drawbacks of each standalone system and achieve centimetre-level positioning accuracy. It can also be successfully applied in a GNSS-denied environment.

Only 2D navigation was investigated in the current platform configuration. In future work, two LiDARs will be installed vertically and horizontally on the mobile platform for 3D position estimation to verify the performance of 3D navigation in more complicated situations. In this paper, we also found that the outputs of a commercial-grade IMU degraded the accuracy of the combined solution in a stationary survey. Several tactical IMUs will be integrated into the current NAVIS setup to evaluate how these higher-accuracy IMUs could benefit indoor mapping accuracy. 

## References

[1] Fotopoulos G., Cannon M. (2001). An overview of multi-reference station methods for cm-level positioning. GPS Solut..

[2] Miller M.M., Soloviev A., Uijt de Haag M., Veth M., Raquet J., Klausutis T.J., Touma J.E. (2010). Navigation in GPS Denied Environments: Feature-Aided Inertial Systems.

[3] Veth M.M., Raquet J. (2007). Fusion of Low-Cost Imaging and Inertial Sensors for Navigation. J. Inst. Navig..

[4] Veth M.J. (2006). Fusion of Imaging and Inertial Sensors for Navigation. Ph.D. dissertation.

[5] Scaramuzza D., Fraundorfer F. (2011). Visual odometry [tutorial]. IEEE Robot. Autom. Mag..

[6] Jiang W., Wang L., Niu X., Zhang Q., Zhang H., Tang M., Hu X. (2014). High-Precision Image Aided Inertial Navigation with Known Features: Observability Analysis and Performance Evaluation. Sensors.

[7] Nützi G., Weiss S., Scaramuzza D., Siegwart R. (2011). Fusion of IMU and vision for absolute scale estimation in monocular SLAM. J. Intel. Robot. Syst..

[8] Lupton T., Sukkarieh S. Efficient integration of inertial observations into visual SLAM without initialization. Proceedings of the IEEE/RSJ International Conference on Intelligent Robots and Systems.

[9] Kneip L., Martinelli A., Weiss S., Scaramuzza D., Siegwart R. Closed-form solution for absolute scale velocity determination combining inertial measurements and a single feature correspondence. Proceedings of the IEEE International Conference on Robotics and Automation (ICRA).

[10] Kleinert M., Schleith S. Inertial aided monocular SLAM for GPS-denied navigation. Proceedings of the IEEE Conference on Multisensor Fusion and Integration for Intelligent Systems (MFI).

[11] Klein I., Filin S. (2011). Lidar and INS Fusion in Periods of GPS Outages for Mobile Laser Scanning Mapping Systems. ISPRS-Int. Arch. Photogramm. Remote Sens. Spat. Inf. Sci..

[12] Kohlbrecher S., Von Stryk O., Meyer J., Klingauf U. A flexible and scalable slam system with full 3d motion estimation. Proceedings of the IEEE International Symposium on Safety, Security, and Rescue Robotics.

[13] Soloviev A. Tight coupling of GPS, laser scanner, and inertial measurements for navigation in urban environments. Proceedings of the Position, Location and Navigation Symposium, 2008 IEEE/ION.

[14] Wang X., Toth C., Grejner-Brzezinska D., Sun H. Integration of terrestrial laser scanner for ground navigation in GPS-challenged environment. Proceedings of the XXI Congress of International Society for Photogrammetry and Remote Sensing.

[15] Borges G.A., Aldon M.J. (2002). Optimal Robot Pose Estimation using Geometrical Maps. IEEE Trans. Robot. Autom..

[16] Pfister S.T. (2006). Algorithms for Mobile Robot Localization and Mapping Incorporating Detailed Noise Modeling and Multi-scale Feature Extraction. Ph.D. dissertation.

[17] Horn J.P. (1997). Bahnführung Eines Mobilen Roboters Mittels Absoluter Lagebestimmung Durch Fusion Von Entfernungsbild-und Koppelnavigations-Daten. Ph.D. Thesis.

[18] Giorgio G., Cyrill S., Wolfram B. (2007). Improved Techniques for Grid Mapping with Rao-Blackwellized Particle Filters. IEEE Trans. Robot..

[19] Tang J., Chen Y., Chen L., Liu J., Hyyppä J., Kukko A., Chen R. (2015). Fast Fingerprint Database Maintenance for Indoor Positioning Based on UGV SLAM. Sensors.

[20] Kim H.S., Baeg S.H., Yang K.W., Cho K., Park S. An enhanced inertial navigation system based on a low-cost IMU and laser scanner. Proceedings of the SPIE 8387, Unmanned Systems Technology XIV, 83871J.

[21] Maybeck P.S. (1994). Stochastic Models, Estimation And Control.

[22] Tang J., Chen Y., Jaakkola A., Liu J., Hyyppä J., Hyyppä H. (2014). NAVIS—An UGV Indoor Positioning System Using Laser Scan Matching for Large-Area Real-Time Applications. Sensors.

[23] Jekeli C. (2001). Inertial Navigation Systems With Geodetic Applications.

[24] Titterton D., Weston J. L. (2004). Strapdown Inertial Navigation Technology.

[25] Shin E.-H., Naser E.-S. (2001). Accuracy Improvement of Low Cost INS/GPS for Land Applications.

[26] Shin E.H. Estimation Techniques for Low-Cost Inertial Navigation. http://www.geomatics.ucalgary.ca/links/GradTheses.html.

[27] Kurt K., Ken C. Markov localization using correlation. Proceedings of the Sixteenth International Joint Conference on Artificial Intelligence.

[28] Olson E.B. Real-time correlative scan matching. Proceedings of IEEE International Conference on Robotics and Automation (ICRA).

[29] Simon D. (2006). Optimal State Estimation: Kalman, H Infinity, and Nonlinear Approaches.

[30] Grewal M.S., Andrews A.P. (1993). Kalman Filtering: Theory and Practice.

[31] MTi User Manual. https://www.xsens.com/wp-content/uploads/2013/12/MTi-User-Manual.pdf.

